# A simulation study of the impact of the public–private partnership strategy on the performance of transport infrastructure

**DOI:** 10.1186/s40064-016-2533-6

**Published:** 2016-07-02

**Authors:** Zhengfeng Huang, Pengjun Zheng, Yanqiang Ma, Xuan Li, Wenjun Xu, Wanlu Zhu

**Affiliations:** Faculty of Maritime and Transportation, Ningbo University, Ningbo, China; National Traffic Management Engineering and Technology Research Centre Ningbo University Sub-Centre, Ningbo, China; Jiangsu Province Collaborative Innovation Center for Modern Urban Traffic Technologies, Nanjing, China

**Keywords:** PPP model, Agent simulation, Freeway network

## Abstract

The choice of investment strategy has a great impact on the performance of transport infrastructure. Positive projects such as the “Subway plus Property” model in Hong Kong have created sustainable financial profits for the public transport projects. Owing to a series of public debt and other constraints, public–private partnership (PPP) was introduced as an innovative investment model to address this issue and help develop transport infrastructure. Yet, few studies provide a deeper understanding of relationships between PPP strategy and the performance of such transport projects (particularly the whole transport system). This paper defines the research scope as a regional network of freeway. With a popular PPP model, travel demand prediction method, and relevant parameters as input, agents in a simulation framework can simulate the choice of PPP freeway over time. The simulation framework can be used to analyze the relationship between the PPP strategy and performance of the regional freeway network. This study uses the Freeway Network of Yangtze River Delta (FN-YRD) in China as the context. The results demonstrate the value of using simulation models of complex transportation systems to help decision makers choose the right PPP projects. Such a tool is viewed as particularly important given the ongoing transformation of functions of the Chinese transportation sector, including franchise rights of transport projects, and freeway charging mechanism.

## Background

Public–private partnership (PPP) is a contractual scheme under which public sector and private firms cooperate and share risks and profits to construct infrastructure projects, or provide public products and services. Due to the potential contribution to reduced transaction costs, innovation, continuous exploitation of a learning curve, the re-focusing of government on its core tasks, and the enabling of large infrastructure investments, PPP has been widely applied in projects of transport infrastructure such as roads, rails, airports, seaports, waterways, etc. (Cruz and Marques 2013a; Siemiatycki 2011). Moreover, the recent liberalization in transport sector and global economic crisis are favoring the implementation of transport projects through the PPP model (Tsamboulas et al. 2013). In fact, there are many successful cases such as the “Subway plus Property” model and “Landlord Port” model.

Actually, different models have been developed to implement PPP projects in the field of transportation engineering. Based on the involvement of the private sector and risk allocation between the public and private sector, the models can be classified into 12 types and grouped further into 4 categories, including operations and maintenance, concession (public ownership of the facilities), concession (private ownership of the facilities) and full privatization (Percoco 2014). The private sector can get involved in a transport PPP project at different phases such as the very beginning of design, construction, financing, operation or maintenance, even through the whole project lifecycle. Some PPP models such as Build-Operate-Transfer (BOT) and Build-Own-Operate (BOO) focus on the construction quality of the transport projects. The Sines Container Terminal in Portugal and the Valencia Cruise Terminal in Spain were constructed under this sort of PPP model (Roumboutsos et al. 2013). Some models such as operating concession tend to involve the private sector during the operation phase aiming to improve the service quality. Many PPP projects in the area of public urban transportation like the Line 4 Subway project in Beijing are representatives of this sort (De Jong et al. 2010). Besides, some models such as full/partial privatization or design-build-finance-operate (DBFO) are adopted as a result of the lack of public finance in order to provide transport service in an earlier stage. The M6 Tollway in the UK implemented using the DBFO is one example of this sort (U.S. Department of Transportation 2007). Therefore, various PPP models have been extensively applied in the construction of transport infrastructure. But the strategy, which infrastructure should adopt PPP model, remains an unresolved problem.

If PPP strategy should be made for a transportation system, the users, operators, planners, and owners would constitute a set of distinct stakeholders, with each stakeholder making strategic decisions and investments toward fulfilling its own objectives for system performance. Ultimately, however, transportation system performance is a function of the interactions among and the decisions taken by all stakeholders. These interactions can also complicate efforts at the choice issue of PPP freeway. This paper adopts the approach of agent simulation to acquire the performance of transport infrastructure. The simulated performance can then assess the validity of PPP strategy. The Freeway Network of Yangtze River Delta (FN-YRD) in China was chosen as the simulation object. The PPP strategy can hereby be applied to develop any road sections, bridges or tunnels. Major reasons for the choice of FN-YRD include: (1) The region of FRD is an advanced area in China. It has reached a level of middle-developed countries in terms of GDP and density of road network per capita. Therefore its results are useful to developed countries. (2) The FN-YRD has been developed after China’s “reform and opening” policy by the end of 1980s. But, the regional prosperity was achieved under a political system that is not yet sound. This experience may be valuable for the countries whose political system development is on the match.

## Literature review

The early attempts of using the PPP model to build up transport projects were found in the late 1970s with highway concessions in France and the mid-to-late 1980s in Spain and England. The strongest impetus fostering transport PPP projects occurred in the 1990s in the UK, where economic reforms encouraged a number of efforts to privatize major elements of the national transport systems. Under the name of Private Finance Initiative (PFI), legislative and regulatory reforms were put into place to carry PPP projects primarily focused on the transport infrastructure including railroads, public transportation, and aviation (U.S. Department of Transportation 2007). Since then, the PPP usage spread fast worldwide, first into other developed countries such as many European countries, the US, Australia, Canada etc., later into developing ones in Asia, South America and other regions.

Along with the worldwide adoption of the PPP model into developing transport infrastructure, an increasing number of papers and reports are published. By reviewing the literature, different focuses are found on these researches. Some made efforts in summarizing the critical successful factors of PPP usage in general (Mu et al. 2010; Thomas Ng et al. 2012; Yun et al. 2015) or the impacts of certain factors like the institutional factor (Panayides et al. 2015; Percoco 2014; Verhoest et al. 2015). Some literatures focus on specific sectors of transport area such as airports (Farrell and Vanelslander 2015), ports (Cabrera et al. 2015; Macario 2014), construction (Tang et al. 2010) or urban transport (Willoughby 2013). Other research directions include PPP contract and negotiation (Cruz and Marques 2013b; Domingues and Zlatkovic 2015; Hart 2003; Krüger 2012; Xu 2010), and risk allocation, assessment or mitigation (Chan et al. 2011; Li et al. 2005; Vassallo 2006). Beyond that, a large number of publications are focusing on discussing performance of the transport PPP projects. Compared to the traditional financing styles, PPP projects are proved for having advantages of performing transport services on-time and on-budget, gaining efficiency and effectiveness, decreasing overall costs in construction and operation (Cruz and Marques 2013a; Grimsey and Lewis 2002). An overall success the PPP model in terms of time, cost and quality for multi-stakeholders (public, private and user) was implicated by analyzing four PPP transport projects from four different EU countries using the approach of Qualitative Comparative Analysis (Liyanage and Villalba-Romero 2015). Service quality is even ranked as the most important factor when government consider choose the PPP model (Tsamboulas et al. 2013). In addition, The UK Treasury estimates that the use of PPP model can produce a cost-saving of 17–25 % on average over all sectors (Alfen et al. 2009). Similar results are also provided by evidence from Australia. The PPP model has advantages of cost-saving of 9–23 % and on-time delivery over traditional ones (Infrastructure Partnerships Australia 2007). Transport infrastructure requires a high investment and will increase the burden of public deficit. The PPP model provides an alternative through the involvement of private sector and delivers the transport service faster by avoiding inflationary cost increases (U.S. Department of Transportation 2007). Further, the PPP model fosters innovation. It provides a flexible way to charge transport service tolls. Beside the traditional mileage, other criteria, such as vehicle types in terms of emission volume or size, occupancy level, travel period (peak-time vs. off-peak time), can be used to increase the usage of transport service and avoid congestion and pollution (Tamayo et al. 2014).

Overall, most previous and current publications about the impact of the PPP model on the performance of transport infrastructure are mainly focusing on the construction phase. Few ones are found to analyze the performance after construction.

Further, public investment decisions tend to be made in a short-term. A feasibility study of a PPP project is usually conducted when the transport project is to be initiated. Single transport project has limited economic benefit. The relevant evaluations are not comprehensive and the rationality of decision making is susceptible. Some transport projects that are feasible during the evaluation phase are finally proved to be a failure after implementation. For instance, the PPP project of Hangzhouwan Bridge was a great success at the beginning after construction in 2008 as it reduces the distance between Ningbo and Shanghai by 30 %. Unfortunately, the high-speed railway (HSR) was put into practice and the Jiashao Bridge (a neighboring bridge) was also built up in 2013. Massive travelers have been attracted from using the Hangzhouwan Bridge. As a result, many private investors have to leave the PPP project of Hangzhouwan Bridge. The share belonging to the private sector decreased from above 50 % in 2009 to approximately 15 % in 2013.

Therefore, it is critical for governments to have a comprehensive analysis of scale effect towards a series of transport PPP projects in order to achieve reliable decisions. However, it is quite complicated to evaluate the whole regional freeway network. When examining the performance of the network under the PPP strategy, a large number of factors, including the freeway feature, travel modes, travel behaviors, and the evolution mechanism of other travel modes, should be taken into consideration. Hence, it is significant to establish the evolution model of the performance of transport infrastructure.

This paper adopts agents to simulate the evolution of performance indexes for transport infrastructure. Agent-based modeling methodology has a long lineage, beginning with von Neumann’s (1966) work on self-reproducing automata. Agents are “objects with attitudes” (Bradshaw 1997). The application of agents in transportation field is popular, e.g., traffic control using agent simulation (De Oliveira and Camponogara 2010). However, few researches predict the performance of transport infrastructure by comprehensively considering the complex interaction in the traffic system. We will conduct this kind of research. Besides, although lots researches have focused on the performance of transport infrastructure, most of them do the qualitative analysis and lack the model structure. This kind of research does not show a transplantable character.

The innovative contribution of this paper is as follows: The interaction relationship between the PPP strategy and the applied objects are taken into consideration when doing the feasibility study. That is an improvement compared to the traditional method (conduct single project evaluation solely). The subsequent sections are organized as follows. A dissection analysis of the impact factors of the PPP model on the transport infrastructure is firstly conducted. Based on the analysis, agent-based simulation framework is established to make PPP strategy. The simulation results are then presented and discussed.

## The impact of PPP strategy on the performance of transport infrastructure

**Fig. 1:**
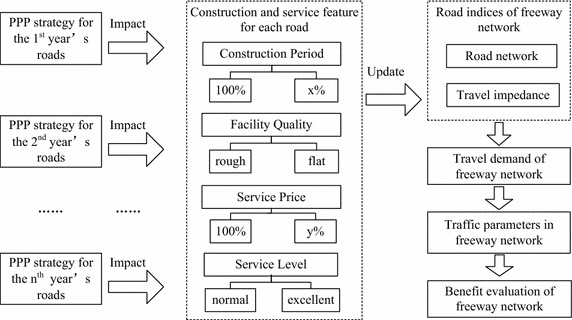
Impact procedure of PPP strategy on the freeway network

Decision maker should understand the impact of PPP strategy on the whole transportation system so as to make a better decision about the investment model for each road. Generally, transportation sectors need to schedule the freeway investment each year. The PPP strategy should also be determined year by year. In our model, the PPP strategy should be determined for each year’s roads. As shown on Fig. 1, *n*th year’s roads means *n* is the start year of the constructed roads. If a specific road is invested by PPP model, its construction and service feature would be impacted. This impact can propagate to the whole network. The network structure and road impedance of the following years would be varied. The changes also occur in the travel demand. Finally, the transportation network performances, including the assigned traffic flow and economic indexes, would be varied.

Specifically, the PPP model can impact the performance of the corresponding road by four factors: construction period, facility quality, service price and service level (see Fig. 1).

- Construction period

Evidence from actual transport PPP projects indicates that the PPP model has the advantage of reducing the construction time compared to traditional investment model. The infrastructure facilities can be thereby earlier put into operation to meet the travel demand. The values of construction period presented in Fig. 1 represent the possibility of accomplishing the PPP project. For instance, setting x = 75 means that the PPP model can reduce the construction period to 75 % of the one under traditional investment model.

- Facility quality

One advantage of the PPP model is that both public and private sectors attach great importance to the overall benefits of transport projects. Therefore, sufficient funding will be arranged for the whole project lifecycle through construction to maintenance. On the other hand, we generally find some phenomena under traditional investment model, such as corruption of project funds. That will lead to the waste of finance and human resources, consequently impact the construction quality. The road quality is one critical factor impacting a user’s decision. Given two freeways in parallel, travelers will undoubtedly choose the one with better condition rather than the one full of potholes when other conditions are the same.

- Service price

Price is the direct factor influencing people’s travel demand. In general government has a fixed and unified charge mechanism towards the freeway, which neither increases the travel demand nor improves operation benefits. In fact, the freeways in some developing countries such as China don’t have similar issues in the USA—traffic congestion. The freeways in major regions in China have massive capacity to serve potential travel demand. It may attract more price-sensitive passengers by setting up flexible charging fees. Besides, the HSR has a comparative advantage in long distance. If the freeway doesn’t have any effective measures relating to the charging fees, it will certainly lose market share in the long-distance travel. The value of service price presented in Fig. 1 represents the possibility of charging the users. For instance, setting y = 90 means that the PPP model can reduce the charging fee to 90 % of the one under traditional investment model.

- Service level

Service level is defined as convenience and comfort brought by the whole travel environment to the drivers. If a road can have standard road design, and provide timely information such as work-zone information and dynamic traffic flows, people would have stronger tendency to travel on this freeway.

In fact, there are a good number of publications focusing on the quantitative benefits of the PPP model. 75 % of the British PPP projects have reached and even beyond the requirements in terms of price and quality, and saved 17 % costs. Further, 80 % PPP projects were accomplished on time, compared to 30 % under traditional investment model. 80 % PPP projects could be finished within the planed budget, compared to 25 % under traditional ones. Chile is one leading country using the PPP model to develop public services. Among the whole 36 PPP projects since 1994, 24 ones were used to develop transport infrastructure. The annual investment ranges from 0.3 to 1.7 billion US dollar. By reviewing international publications in the field of transport PPP projects (Alfen et al. 2009; Infrastructure Partnerships Australia 2007; Liyanage and Villalba-Romero 2015; Tamayo et al. 2014; Tsamboulas et al. 2013; U.S. Department of Transportation 2007), it is found that the following features of PPP model are popular: construction period (75 %), facility quality (flat), service price (90 %), service level (excellent). In order to test the validity of PPP strategy in FN-YRD, the aforementioned parameters of PPP model are taken as input for simulation model. The traditional investment model is used as a reference. Their features are as follows: construction period (100 %), facility quality (rough), service price (100 %), service level (normal).

## Simulation framework

**Fig. 2:**
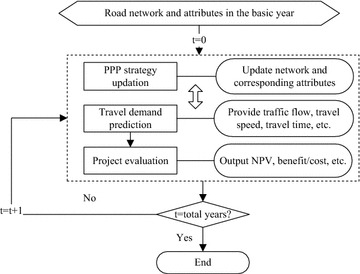
Model structure

The simulation model is conceptually an agent-based model with four components: an environment, rules, agents, and outputs. The simulation comprises three modules (travel demand prediction module, project evaluation module, and PPP investment strategy module), which are illustrated in Fig. 2 and described below. The environment is reflected in the travel demand prediction module. It is represented by the population of each city in YRD, the FN structure under different investment strategy, and travel demand. Rules are embedded in the project evaluation module and the PPP investment strategy module to calculate indexes and implement decision respectively. Agents include government, private enterprise and road user. Outputs include a set of results of the investment decision of PPP model on the FN.

The freeway has a history over 30 years in China. However, the HSR has been put into practice just since 2004. In order to investigate the development of freeway under the influence of HSR, 10 years (2005–2014) was chosen on the basis of the availability of data. Each run through the aforementioned modules represents 1 year. Strategy module is used to update the investment model for the constructed roads in the studied year. The varied strategies would correspond different road network and road attributes in the future. Actually, under the assumed PPP strategy, we could implement the travel demand prediction and project evaluation modules to get the required network performance. This performance can be used to evaluate and update the current PPP strategy in this year conversely. This inner loop procedure would never stop until the network performance can satisfy the requirement of PPP strategy. At the outer loop, the year would increase gradually. The following subsections discuss each module in detail.

### Travel demand prediction module

This module predicts the volume of traffic flow on each link in FN-YRD for each year in the simulation. This procedure is accomplished with a simplified aggregate four-step model, with trip generation and distribution based on a traditional gravity function of formula (1). The variables in numerator represent the number of private cars, as most travel demand is generated by car owners. The denominator is represented by a weighted sum of distance, price, facility quality and service level of the shortest inter-city freeway. The qualitative values are defined in a 3-point scale. As to the construction period under different investment models, it is already reflected on the changed schedule of road opening.

1 $$T_{ij} = a\frac{{P_{i} P_{j} }}{{C_{ij}^{b} }}$$

where, *T*_*ij*_: total number of trips between municipality *i* and *j*; *P*_*i*_: number of private cars of municipality *i*; *P*_*j*_: number of private cars of municipality *j*; *C*_*ij*_: weighted sum of distance, price, facility quality and service level of the shortest inter-city freeway; *a* and *b*: parameters.

Considering the competition of newly opened HSR, a part of travel demand on freeway may be extracted. The remaining demand is calculated as:

2 $$R_{ij} = T_{ij} \frac{{\exp \left( { - \theta C_{ij} } \right)}}{{\exp \left( { - \theta C_{ij} } \right) + \exp \left( { - \theta D_{ij} } \right)}}$$

where, *D*_*ij*_: travel cost of HSR. It is weighted sum of travel fee, travel time, station access time. *θ*: a positive parameter.

Table 1 presents an inter-city travel mode choice comparison between HSR and freeway. The results are generated based on the mathematical model and investigation separately. A non-significant difference indicates the reliability of the mathematical model.

**Table 1:** Inter-city travel mode choice between HSR and freeway

Factor	Hangzhou-Shaoxing	Ningbo-Shaoxing	Nanjing-Huzhou
Distance (km)	40	110	220
Travel fees for HSR (¥)	19.5	51.5	85
Travel time by HSR (m)	20	40	60
Station access time (m)	40	30	40
Ratio of choosing freeway based on simulation (%)	89	73	67
Ratio of choosing freeway based on investigation (%)	92	72	61

Remarks: the fee to use freeway including toll and fuel is generally ¥ 1/km; “m” stand for minute

As indicated earlier, all trips are assigned to the FN, an assignment that reflects the dominance of the auto mode for intercity travel in YRD. Finally, trips are assigned to the path by use of an incremental assignment approach. This method gets a result approximate to that of equilibrium traffic assignment. It follows the principle that traveler’s priority route is the shortest freeway. Only if it is capacity constrained, the second shortest route is under consideration.

### Project evaluation module

Net Present Value (NPV) is used to evaluate the effectiveness of the PPP strategy. NPV is defined as the difference between the present values of incoming and outgoing cash flows over a period of time. This method has taken the operation benefits and the internal rate of investment return into consideration; thus it complies with the requirement of financial effectiveness evaluation. Therefore, it is able to use the NPV calculation formula (3) to evaluate the PPP strategy.

3 $$NPV = \sum\limits_{i = 1}^{n} {[(CI_{p} - CO_{p} )(1 + m)^{ - i} ]}$$

where *n*: total period of the investment style; *CI*_*p*_, *CO*_*p*_: represent incoming and outgoing cash flows of each year of operation sectors; *m*: discount rate.

### PPP strategy module

Mathematically, PPP strategy is a selection problem with 2^*ρ*^ possible combinations. The variable *ρ* herein represents the amount of planned roads. Just 30 roads would take the calculation counts of travel demand prediction and project evaluation by billions. Therefore, we design a heuristic method to solve this problem rapidly. The PPP strategy is solved year by year in our method. The year-by-year method is practical, because we do not know the road planning of future. For instance, if we need to determine a PPP strategy for the constructed road this year, we cannot consider the impact of the roads which may be planned in future. In addition, we make an assumption when calculating NPV. Except for the current and previous years, the planned roads in the future years are assumed not to be considered. Based on the calculated NPV, the PPP strategy of current year could be updated by several rules. Then, we would come back to the step of NPV calculation. This iteration procedure continues until the updated PPP strategy in the current year can meet the requirement. Our case application would validate this method. The following sections would be used to set the PPP strategy of the current year.

PPP investment decisions are results of interactions of multi-agents. The government makes decision under the constraint of public budget and aim of chasing the profit of freeway projects. The private enterprises make decision on the basis of internal rate of return. Users’ requirement bases on the comprehensive travel cost. To some extent, both government and private enterprises prefer to freeway projects with higher profit rate rather than the ones featuring with negative NPV. In terms of project with negative PNV, the public welfare function forces government to undertake the construction responsibility of freeway projects with negative NPV. In terms of project with positive NPV, government usually tends to operate the ones with higher profits and transfer the ones with lower profits, because the latter ones may have a risk of operating at a loss. In user’s view, they expect all the freeway services are operated by private enterprises so as to enjoy high operation quality. Considering all the interests of three agents, the private enterprises should take the low-profit projects and the government needs to transfer parts of high-profit projects to private enterprises. This principle is used to make the PPP strategy for the current year’s projects *A*:

1. Step 1, each project within *A* is assumed to be invested by PPP model.
2. Step 2, predict the incoming and outgoing cash in the operation years; obtain the NPV for projects within *A*; if step 1 is not the front step, turn to step 4.
3. Step 3, projects with positive NPVs are selected each year and ranked from low to high as alternatives for the PPP investment decisions; the front 80 % of all the alternative projects each year will be chosen for the PPP model; the remaining projects are for traditional investment, turn to step 2.
4. Step 4, if each PPP project is profitable, end this year’s simulation and turn to next year’s simulation; otherwise, transfer the unprofitable projects to traditional investment, turn to step 2.

## Results

### Input data

YRD is taken as the simulation object. The input data are presented in Table 2. The start and end years are 2005 and 2014 respectively. For the design speed, most of the freeway in China is limited by 100–120 km/h. Three intervals of design speed are set in this case. When vehicle density is the same, traffic capacity would correspond to the design speed. Referring traffic engineering manual, the corresponding traffic capacities are listed in Table 2. The parameters of travel demand prediction refers to the traffic planning experience in Chinese cities. The parameters in project evaluation are explained as follows. The discount rate reflects the time value of cash. The traveler value of time means how much does the time deserves. It is investigated by survey. The peak-hour traffic in peak direction reflects asymmetrical distribution of traffic flow on a road. Ratio of traffic in peak hour reflects the amount of traffic flows in peak hour. By the end of each year (each simulation run), the attributes such as the FN and OD matrix will be updated for the next step simulation. This process continues until the end of the 10th year. Relevant data will then be produced for deeper analysis.

**Table 2:** Variables and values used in simulation model

Module	Variables	Values
General	Start year	2005
	End year	2014
FN	Design speed: low	100 km/h
	Design speed: middle	110 km/h
	Design speed: high	120 km/h
	Traffic capacity: low-speed	2000 pcphpl
	Traffic capacity: mid-speed	2200 pcphpl
	Traffic capacity: high-speed	2400 pcphpl
Travel demand prediction	*a*	0.005
	*b*	1.6–2.8 (depending on the sample data)
	*θ*	0.5
Project evaluation	Evaluation time horizon	10 years
	Discount rate	5 %
	Traveler value of time	¥ 50/h
	Peak-hour traffic in peak direction	65 %
	Ratio of traffic in peak hour	13.5 %

### Output data

**Fig. 3:**
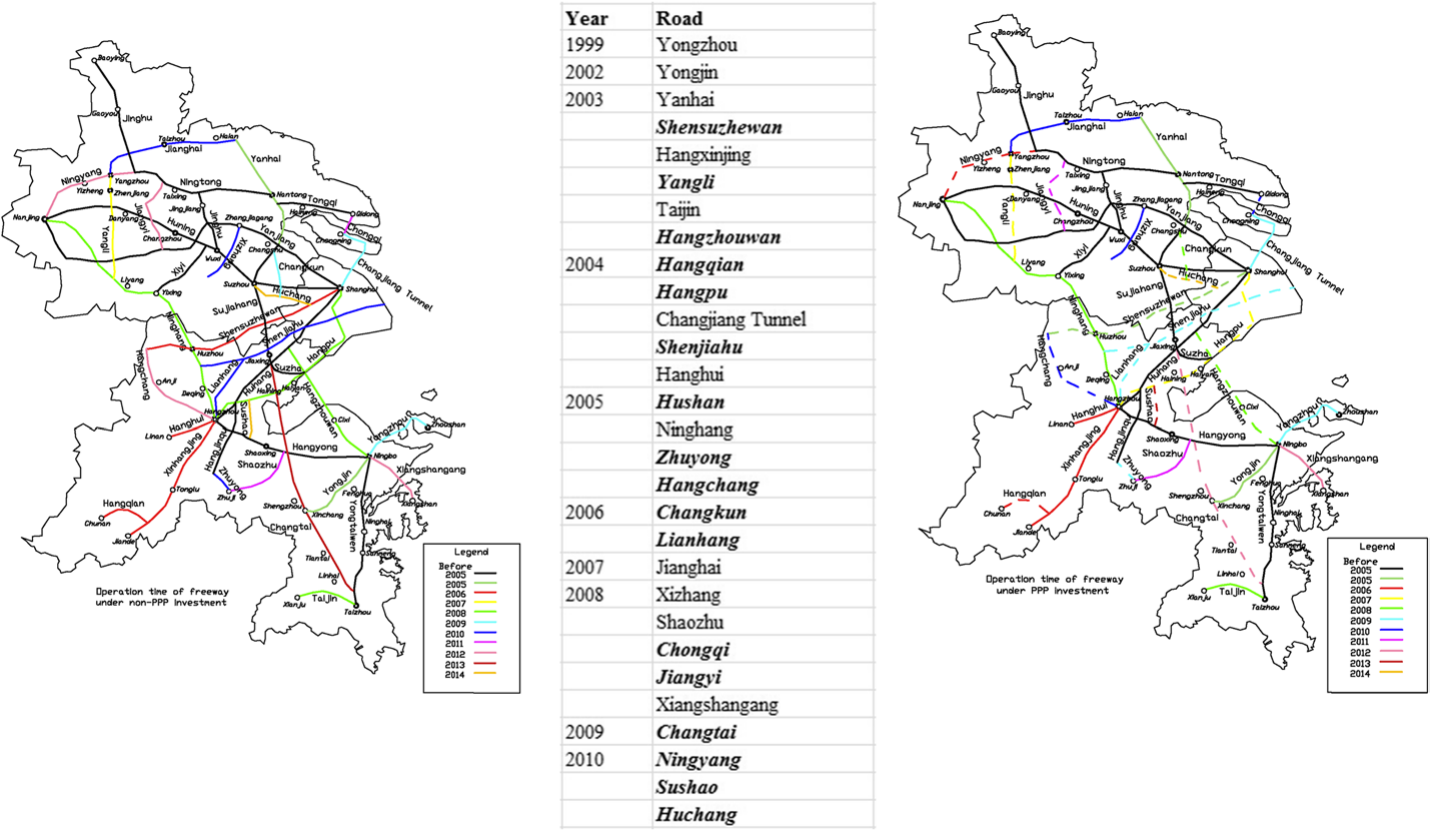
Opening time of FN-YRD under different investment strategies

Figure 3 gives an opening time comparison of the FN in YRD under the traditional investment strategy and the PPP strategy. The start year for construction of each road is attached in the middle of the figure. Roads labeled bold and italic are chosen for the PPP model. These roads are sketched with dotted lines in the right figure.

The output data are summarized in Table 3 and provide a comparison of the performance between the PPP strategy and the traditional one. The PPP strategy has a comparative advantage in terms of most performance indexes, which indicates the feasibility of adopting the PPP model into transport projects. An exception is the average travel time. The reason why travel time is longer under the PPP strategy is that the travel distance is increased. It also indicates that the travel convenience is improved under the PPP strategy, thus people prone to increase their travel distance. NPV in the table is a significant evaluation metric, and would be analyzed in detail in the following sections.

**Table 3:** Performance metrics for PPP strategy and traditional strategy

Performance metric	Results of PPP strategy	Results of traditional strategy
Number of trips per day (2014)	1470,000	1260,000
Average travel distance (2014)	195 km	156 km
Average peak travel time (2014)	1.86 h	1.61 h
Average peak speed (2014)	105 km/h	97 km/h
Average daily total delay (2014)	410,000 h	467,000 h
Total investment (2005–2014)	268 bil. CNY	224 bil. CNY
NPV	109.9 bil. CNY	87.3 bil. CNY

### Analysis of demand change under PPP strategy

**Fig. 4:**
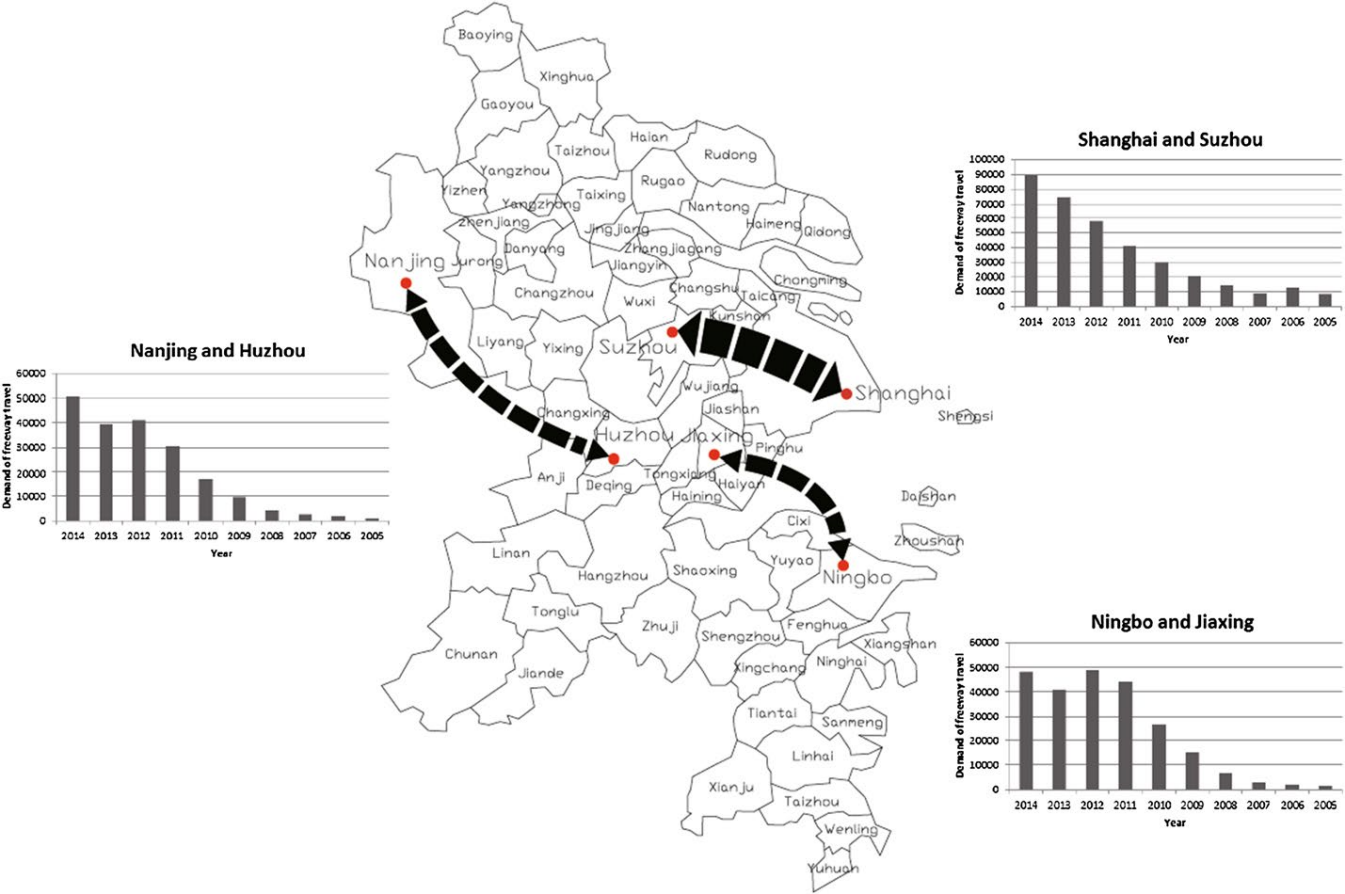
Inter-county map and travel demand change between typical counties

Figure 4 shows the travel demand change between typical counties, which are connected by both HSR and freeway. Overall, the travel demand has increased remarkably. The reason is that the car ownership in YRD has experienced a high-growth period since 2005. Further, the years of 2007 and 2013 catch specific attentions in terms of travel demand development. Because the high-speed railway was put into operation, which attracted a part of previous vehicle travelers.

There is a temporary demand decline between Shanghai and Suzhou, when the high-speed railway between Shanghai and Nanjing^1^ was put into operation in 2007. However, the travel demand soon increased steadily, because the freeway has a comparative advantage over railway in short travel distance (80 km). Besides, the fast increase in travel demand could also attribute to the auto plate auction. The auction price for license plates boomed recently in Shanghai, which make many Shanghai workers choose to buy cars and live in Suzhou. Subsequently, large traffic volumes are formed between these two cities.

The travel demand between Ningbo and Jiaxin was not large a few years ago. The cargo traffic accessing to Ningbo Port played a significant role. The Hangzhouwan Bridge operated in 2008 improved the traffic accessibility and increased the travel demand, as it shortens the travel distance from 180 to 120 km. But the Jiashao Bridge, a neighboring bridge crossing Hangzhou Bay, operated in 2013, has a great impact on the strengths of Hangzhouwan Bridge in terms of cost and time. In addition to that, high-speed railway was operated between these two cities in 2013. Both result in the declining of car traffic instead of rising. It indicates the risk of recouping freeway construction cost would increase when HSR appears. Only those freeways (e.g., Hangyong Freeway) that have been operated for a long period could recoup the investment. Nowadays alternative travel modes bring fierce competition to the travel demand of freeway, so as to impact its benefits. Therefore, it is critical to consider these impacts when making investment decisions.

Before 2008, people should drive 300 km of freeway to travel from Nanjing to Huzhou. Although there is a direct provincial highway connecting the two cities with a shorter distance, few travelers chose to take this option because of a large number of signal controlled intersections. It would increase travel time and raise the risk of traffic accident. Luckily, the direct freeway was operated in 2008. It reduces the distance to 200 km and increases the travel demand significantly. Besides, although the HSR operated in 2013 has impacted the growth rate of vehicle travelling, its demand is still increasing steadily, which differs from the Hangzhouwan Bridge. One possible reason is that the Tai Lake between Nanjing and Huzhou makes it impossible to choose other bypassing lines.

### Evaluation of investment benefit

**Fig. 5:**
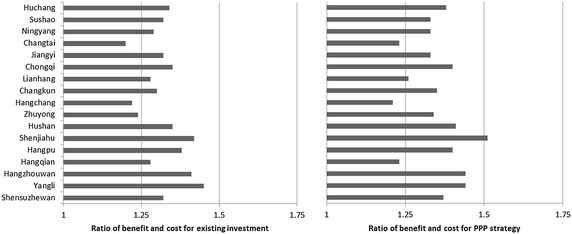
Value of benefit/cost on each freeway of different investment strategies

Following the step of calculating NPV for each freeway, the benefit/cost on each freeway of different investment models is presented on Fig. 5. Most freeways have a higher benefit/cost ratio under PPP investment. In addition to the traditional strengths of the PPP strategy, one external reason plays a critical role. The PPP strategy shortens the construction period of freeways and thus brings forward the date for operation. This advantage reduces the impacts of external factors such as the HSR, because it could race against time to recouping freeway construction cost.

## Conclusions

This paper defines the research scope to a regional freeway network. The impacts of PPP strategy on the performance of transport infrastructure are analyzed. The approach of agent-based simulation is introduced to examine the relationship between the PPP strategy and performance of a freeway network. Further, this paper compares the results with the traditional investment strategy by FN-YRD in China. Some conclusions are achieved. For instance, the PPP strategy can indeed increase the benefit of the whole freeway network. The conclusions can be referred for the application research in the transport PPP projects. Besides, our future focuses are as follows.

1. The prediction accuracy of the traditional gravity four-step model may be not high. Given sufficiently detailed data in future, the activity-based prediction method could be borrowed to achieve accurate travel demand.
2. The method of NPV used to evaluate the PPP strategy doesn’t take the impact of risks into consideration. This needs to be improved in future research.
3. The method choosing freeway to use PPP model is practical but also rough. To make a more scientific decision, it is significant to use the equilibrium theory to generate a general formula in future work.

## Footnotes

1 Suzhou is one stop along the high-speed railway between Shanghai and Nanjing.
